# Insulin Resistance Modifies the Glycemic Impact of Carbohydrate Across Distinct Meal Contexts: A Repeated-Measures Dual-CGM Study

**DOI:** 10.3390/metabo16090671

**Published:** 2026-09-11

**Authors:** Buket Yilmaz Bulbul, Burak Andac, Mehmet Celik

**Affiliations:** Department of Endocrinology and Metabolism, Trakya Faculty of Medicine, Trakya University, Edirne 22030, Türkiye; burakandac@trakya.edu.tr (B.A.); mehmetcelik@trakya.edu.tr (M.C.)

**Keywords:** insulin resistance, HOMA-IR, continuous glucose monitoring, postprandial glycemia, carbohydrate, personalized nutrition, precision nutrition, CGMacros

## Abstract

**Highlights:**

**What are the main findings?**

• Insulin resistance significantly amplified the postprandial glycemic effect of carbohydrate during two distinct structured meal contexts.
• The interaction was supported by a second simultaneously worn CGM device and multiple sensitivity analyses but was not evident during self-selected dinner meals.

**What are the implications of the main findings?**

• The glycemic impact of a given carbohydrate increment may depend partly on the host insulin-resistance phenotype rather than on carbohydrate quantity alone.
• Under self-selected dinner conditions, carbohydrate quantity alone was less informative; this finding should be interpreted cautiously because meal heterogeneity, timing, exposure quality, and reporting variability may all contribute.

**Abstract:**

**Background/Objectives:** Postprandial glycemic responses vary markedly among individuals, but it remains unclear whether the glycemic effect of incremental carbohydrate exposure is systematically modified by insulin resistance. We examined whether insulin resistance alters the carbohydrate–postprandial glucose dose-response across distinct meal contexts. **Methods:** We performed a secondary repeated-measures analysis of the publicly available CGMacros cohort. Forty-five adults spanning normoglycemia, prediabetes, and type 2 diabetes wore blinded FreeStyle Libre Pro and Dexcom G6 Pro continuous glucose monitors while recording meals for approximately 10 days. The primary outcome was 2-h incremental area under the glucose curve (iAUC). Mixed-effects models tested carbohydrate × HOMA-IR interactions, adjusted for protein, fat, fiber, premeal glucose, age, sex, and BMI. Breakfast was the primary structured context, lunch provided within-cohort contextual confirmation, dinner was a free-living contrast, and Dexcom analyses assessed cross-device robustness. **Results:** The primary Libre analysis included 423 breakfasts and 414 lunches from 44 participants. Higher HOMA-IR significantly amplified the iAUC associated with each 10-g increment in carbohydrate during breakfast (interaction β = 178.5 mg·min/dL per 1-SD higher HOMA-IR; 95% CI 79.5–277.5; *p* < 0.001) and lunch (β = 109.8; 95% CI 34.2–185.3; *p* = 0.004). The direction was supported with Dexcom during breakfast (β = 165.3; 95% CI 45.0–285.6; *p* = 0.007) and lunch (β = 87.9; 95% CI 2.6–173.1; *p* = 0.043) and remained robust in GEE, participant fixed-effects, random-slope, activity-adjusted, and other sensitivity analyses. No positive interaction was observed during dinner. **Conclusions:** In this observational secondary analysis, insulin resistance modified the glycemic impact of carbohydrate across two structured meal contexts, with supportive cross-device evidence. These findings are hypothesis-generating and do not establish HOMA-IR-based dietary prescriptions; prospective external validation is required.

## 1. Introduction

Postprandial glycemia is increasingly recognized as a dynamic component of metabolic health that is not fully captured by fasting glucose or glycated hemoglobin. Continuous glucose monitoring (CGM) studies have demonstrated substantial interindividual variation in glycemic responses to apparently similar foods and meals. Large-scale work by Zeevi et al. and the PREDICT consortium established that both meal composition and person-specific characteristics contribute to this heterogeneity [1,2]. More recently, deep metabolic phenotyping has linked differential glycemic responses to standardized carbohydrate challenges with underlying physiology, including insulin resistance and beta-cell function [3].

Despite these advances, much of the precision-nutrition literature has focused on predicting the magnitude of postprandial glycemic responses rather than estimating how a specific meal component interacts with the metabolic phenotype of the host. Carbohydrate quantity is a major determinant of postprandial glycemia, but the same carbohydrate increment may not have an equivalent glycemic effect in individuals with different degrees of insulin resistance. This distinction is clinically relevant because an interpretable host–meal interaction may offer information that is complementary to black-box prediction and conventional glycemic classification.

The recently released CGMacros dataset provides a unique opportunity to study this question. CGMacros contains repeated meal-level macronutrient information, blinded recordings from two simultaneously worn CGM systems, physical-activity data, and fasting metabolic measures in 45 adults spanning normoglycemia, prediabetes, and type 2 diabetes [4]. Breakfast and lunch were deliberately designed to span distinct macronutrient compositions, whereas dinner was self-selected, allowing structured and free-living meal contexts to be examined within the same participants [4].

We therefore investigated whether insulin resistance, estimated by the homeostasis model assessment of insulin resistance (HOMA-IR) [5], modifies the association between meal carbohydrate content and the 2-h postprandial glucose response. We designated breakfast as the primary structured discovery context and lunch as a second structured meal context for within-cohort contextual confirmation, assessed Dexcom as supportive cross-device robustness, and used self-selected dinner meals as a free-living contrast. We further compared HOMA-IR with HbA1c, the triglyceride/HDL ratio, and BMI as candidate metabolic effect modifiers.

## 2. Materials and Methods

### 2.1. Data Source and Study Population

This study was a secondary analysis of the publicly available CGMacros dataset (version 1.0.0), accessed through PhysioNet. The original study recruited participants at the Sansum Diabetes Research Institute in Santa Barbara, California, between 2021 and 2024. Participants provided informed consent under Advarra IRB protocol Pro00049227 (ClinicalTrials.gov NCT04991142) [4]. According to the original protocol, participants with type 2 diabetes who were receiving insulin, an injectable GLP-1 receptor agonist, or oral glucose-lowering medication other than metformin were excluded [4]. Participant-level metformin exposure was not available in the public files used for the present analysis. Forty-five participants completed the study. For this secondary analysis, glycemic-status groups followed the published HbA1c-based categorization: 15 with normoglycemia (HbA1c < 5.7%), 16 with prediabetes (HbA1c 5.7–6.4%), and 14 with type 2 diabetes (HbA1c > 6.4%) [4]. The public dataset did not provide a separate variable allowing independent adjudication of the clinical diabetes diagnosis.

At baseline, the dataset included age, sex, BMI, HbA1c, fasting glucose, fasting insulin, triglycerides, total cholesterol, HDL-C, LDL-C, and related metabolic measurements. Participants wore two blinded CGM devices simultaneously: an Abbott FreeStyle Libre Pro sensor (Abbott Diabetes Care Inc., Alameda, CA, USA) and a Dexcom G6 Pro sensor (Dexcom, Inc., San Diego, CA, USA). A Fitbit Sense device (Fitbit, Inc., San Francisco, CA, USA) was used to capture physical activity. The publicly released dataset provides linearly interpolated one-minute CGM and activity time series [4].

### 2.2. Meal Contexts and Meal-Level Quality Control

Participants recorded food intake for approximately 10 consecutive days. Breakfasts consisted of protein shakes designed to provide varying amounts of carbohydrate, protein, fat, and fiber. Lunches were selected from predefined meals from a fast-casual restaurant and were likewise designed to span a range of macronutrient compositions. Dinners were self-selected. Participants were instructed to separate breakfast and lunch, and lunch and dinner, by at least 3 h [4].

Each timestamped eating event was extracted from the participant time-series files. Snacks were retained for event counting and for identifying overlap but were not included in the main meal-context analyses. A breakfast, lunch, or dinner was excluded from the 2-h analysis if another eating event occurred within 120 min after meal initiation or if carbohydrate, protein, fat, or fiber information was incomplete. For each CGM platform, a complete 11-min premeal window from −15 to −5 min was required. The 2-h postmeal window required at least 80% CGM coverage (≥97 of 121 one-minute time points).

### 2.3. Postprandial Glycemic Outcomes

Premeal glucose was defined as the median CGM value from −15 to −5 min relative to meal initiation. The primary outcome was the 2-h incremental area under the glucose curve (iAUC0–120), calculated by trapezoidal integration of glucose values relative to the premeal baseline using the available time stamps from 0 to 120 min. Negative excursions below baseline were retained rather than truncated. Peak glucose excursion, defined as the maximum 0–120-min glucose value minus the premeal baseline, was examined as a secondary outcome.

The Libre-derived iAUC was selected as the primary outcome platform because the original CGMacros technical validation used Libre for the principal breakfast iAUC analyses [4]. Dexcom-derived outcomes were analyzed separately as supportive cross-device robustness analyses because systematic differences between simultaneously worn CGM systems have been described both in CGMacros and in prior studies [4].

### 2.4. Metabolic Exposures and Covariates

HOMA-IR was calculated as fasting insulin (µU/mL) × fasting glucose (mg/dL)/405 [5]. The triglyceride/HDL-C ratio was calculated from fasting lipid measurements. HOMA-IR, HbA1c, TG/HDL-C, and BMI were standardized to z scores before interaction modeling so that interaction estimates represent the additional iAUC associated with a 10-g carbohydrate increment per 1-SD higher metabolic marker. Protein, fat, fiber, premeal glucose, age, sex, and BMI were included as covariates in the primary HOMA-IR models. Mean postmeal METs from 0 to 120 min were added in a sensitivity analysis.

### 2.5. Statistical Analysis

Continuous baseline variables are summarized as median [interquartile range] and categorical variables as n (%). Baseline comparisons across glycemic-status groups used Kruskal–Wallis tests for continuous variables and the Pearson chi-square test for sex. The primary hypothesis was tested using linear mixed-effects models with a participant-specific random intercept. For each meal context, the model included carbohydrate (scaled per 10 g), standardized HOMA-IR, their interaction, protein, fat, fiber, premeal glucose, age, sex, and BMI. The primary discovery analysis used Libre breakfast meals; the same model was then applied to Libre lunch meals as a second structured context for within-cohort confirmation. Dinner was analyzed as a prespecified free-living contrast, and Dexcom models were analyzed separately to assess supportive cross-device robustness.

Robustness analyses included generalized estimating equations with an exchangeable working correlation clustered by participant, participant fixed-effects models, restriction to fully consumed meals, additional adjustment for mean postprandial physical activity, and peak glucose excursion as an alternative outcome. We additionally fitted mixed models with a participant-specific random slope for mean-centered carbohydrate to allow individual carbohydrate-response slopes. Sensitivity analyses also excluded all participants with type 2 diabetes and, where activity data were available, adjusted for meal start time and mean activity during the preceding 60 min. HbA1c, TG/HDL-C, and BMI were evaluated as secondary effect modifiers using the same interaction framework. For the six secondary Libre interaction tests across breakfast and lunch, the Benjamini–Hochberg procedure was used to control the false-discovery rate; HOMA-IR was excluded from this correction because it was the prespecified primary exposure. Because the available cohort size was fixed by the public dataset, no prospective sample-size calculation was performed; effect-size precision was therefore emphasized using 95% confidence intervals. For the null dinner analyses, an 80% minimum detectable interaction was estimated from the model standard error as (z0.975 + z0.80) × SE, and additional sensitivity analyses considered meal timing, pre-dinner activity, central carbohydrate support across HOMA-IR strata, fully consumed meals, and plausibility restrictions based on the accompanying PhysioNet data dictionary. Two-sided *p* < 0.05 was considered statistically significant. Analyses were performed in Python 3.13 using pandas 2.2.3, NumPy 2.3.5, SciPy 1.17.0, and statsmodels 0.14.6.

### 2.6. Use of Generative Artificial Intelligence

Generative artificial intelligence tools were used to assist with data-processing code generation, figure preparation, and language editing. All data transformations, statistical outputs, figures, and references were checked against the source dataset and verified by the authors, who take full responsibility for the content of the manuscript.

## 3. Results

### 3.1. Participant and Meal Characteristics

The study included 45 participants, comprising 15 individuals with normoglycemia, 16 with prediabetes, and 14 with diabetes. The median age was 51 years [IQR 40–58], and 29 participants (64.4%) were women. Median BMI was 30.04 kg/m^2^ [26.92–35.92], median HbA1c was 5.9% [5.5–6.9], and median HOMA-IR was 4.24 [2.25–5.08]. HOMA-IR increased from a median of 1.99 [1.11–3.36] in normoglycemia to 4.61 [3.74–5.52] in prediabetes and 5.06 [4.03–6.67] in diabetes (*p* < 0.001). Baseline characteristics are shown in Table 1.

**Figure 1:**
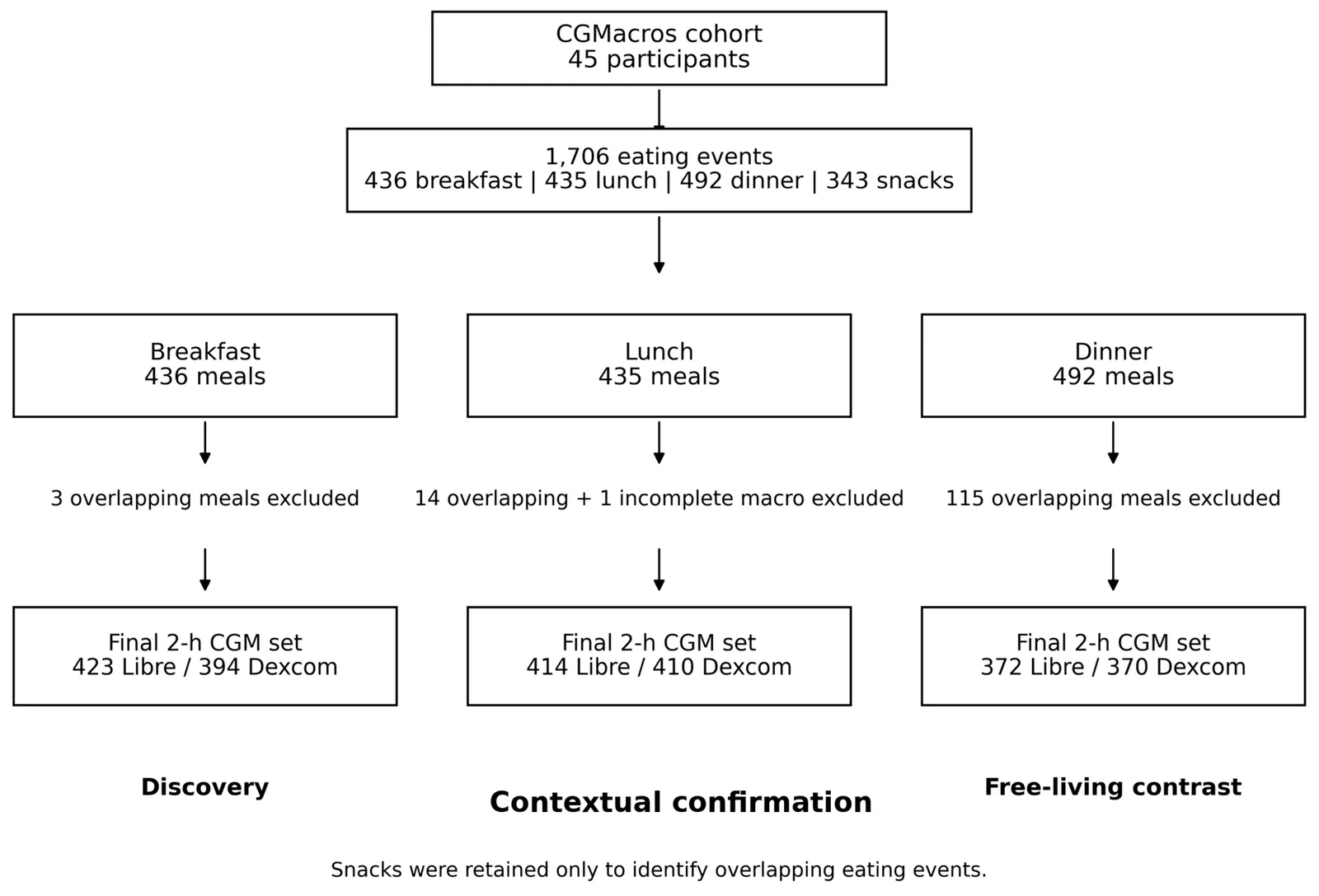
Study flow and derivation of the meal-level analytic sets. Breakfast served as the primary structured discovery context, lunch as a second structured context for within-cohort confirmation, and dinner as the free-living contrast. Snacks were retained only to identify overlapping eating events.

A total of 1706 eating events were identified: 436 breakfasts, 435 lunches, 492 dinners, and 343 snacks. After exclusion of overlapping meals, incomplete macronutrient records, and meal windows without sufficient CGM coverage, the primary Libre analyses included 423 breakfasts and 414 lunches from 44 participants. The corresponding Dexcom analyses included 394 breakfasts and 410 lunches. Dinner analyses included 372 Libre and 370 Dexcom meal episodes (Figure 1). One participant (subject 7) contributed no meal episode that simultaneously satisfied the prespecified premeal baseline and ≥80% 2-h postmeal CGM coverage requirements and therefore did not contribute to the iAUC analytic sets. Carbohydrate exposure was 66 [IQR 66–66] g at breakfast (range 24–73), 76 (40–93) g at lunch (range 16–94), and 44 (22–70) g at dinner (raw range 0–761; 5th–95th percentile 0–131.9). All analytic participants had within-person carbohydrate variation at breakfast and lunch, with median within-participant ranges of 49 and 78 g, respectively. Carbohydrate exposure overlapped closely across lower and higher HOMA-IR strata in the structured contexts: the observed ranges were 24–73 g in both strata at breakfast and 16–94 g in both strata at lunch. Detailed meal composition and exposure-overlap summaries are provided in Table S3.

### 3.2. Insulin Resistance Modified the Postprandial Glycemic Effect of Carbohydrate

In the primary Libre breakfast analysis, higher HOMA-IR significantly amplified the association between meal carbohydrate content and 2-h postprandial glucose iAUC. Each 1-SD higher HOMA-IR was associated with an additional 178.5 mg·min/dL increase in 2-h iAUC for every 10-g increase in meal carbohydrate content (95% CI 79.5–277.5; interaction *p* < 0.001), after adjustment for meal protein, fat and fiber content, premeal glucose, age, sex, and BMI. The interaction was also observed in the separately analyzed lunch context (β = 109.8, 95% CI 34.2–185.3; *p* = 0.004) (Table 2). For clinical interpretability, the 25th and 75th percentiles of HOMA-IR were 2.25 and 5.08. At a 60-g carbohydrate breakfast, holding the remaining model covariates constant, this contrast corresponded to an adjusted predicted 2-h iAUC difference of approximately 2399 mg·min/dL, equivalent to about 20.0 mg/dL in average incremental glucose over 120 min. The interaction component alone accounted for approximately 1005 mg·min/dL of this difference. This model-based illustration is intended to convey effect magnitude and should not be interpreted as a dietary treatment threshold.

**Figure 2:**
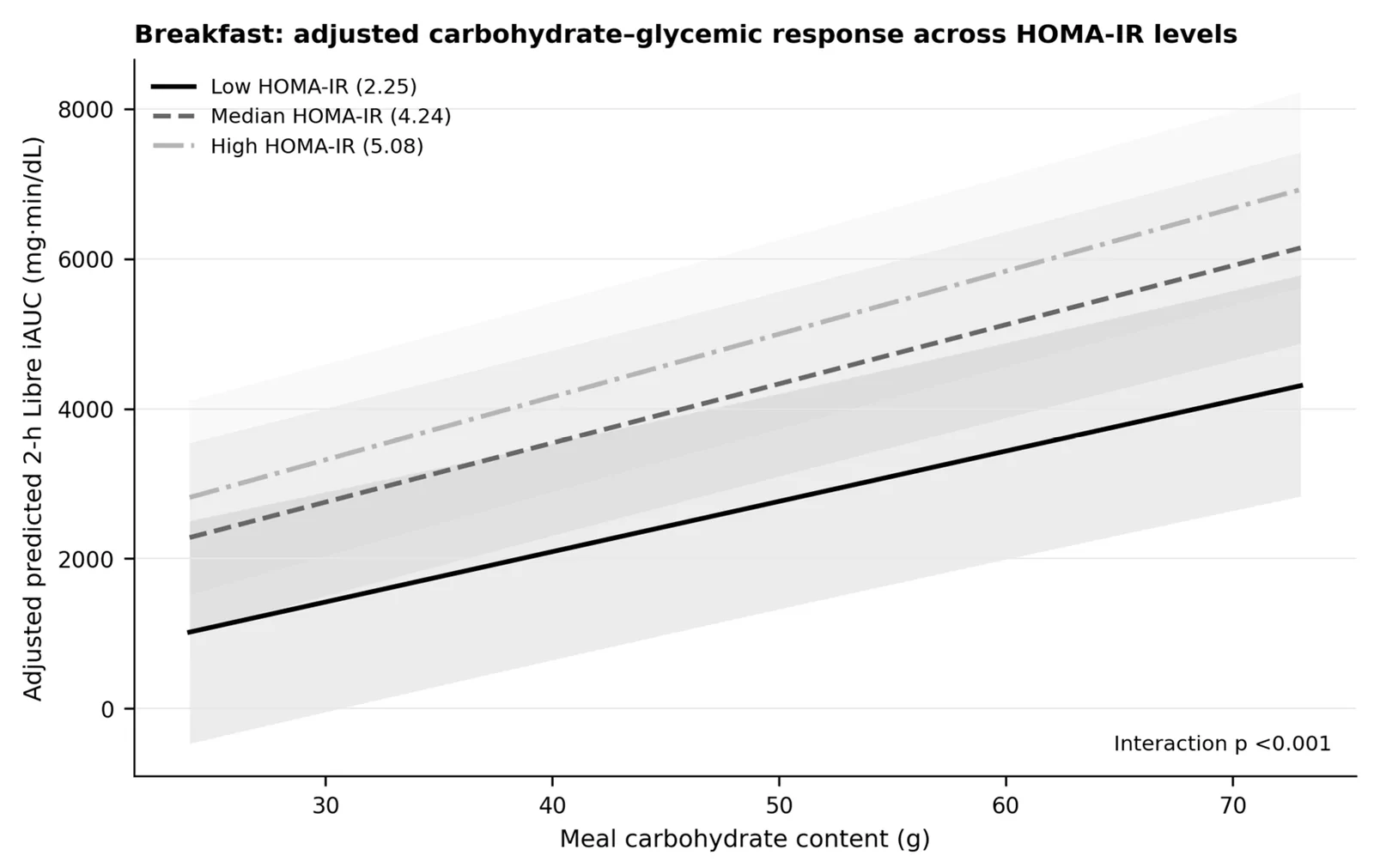
Adjusted predicted Libre 2-h iAUC across meal carbohydrate content at low, median, and high HOMA-IR levels during breakfast. Lines represent model-based estimates and shaded bands indicate 95% confidence intervals.

**Figure 3:**
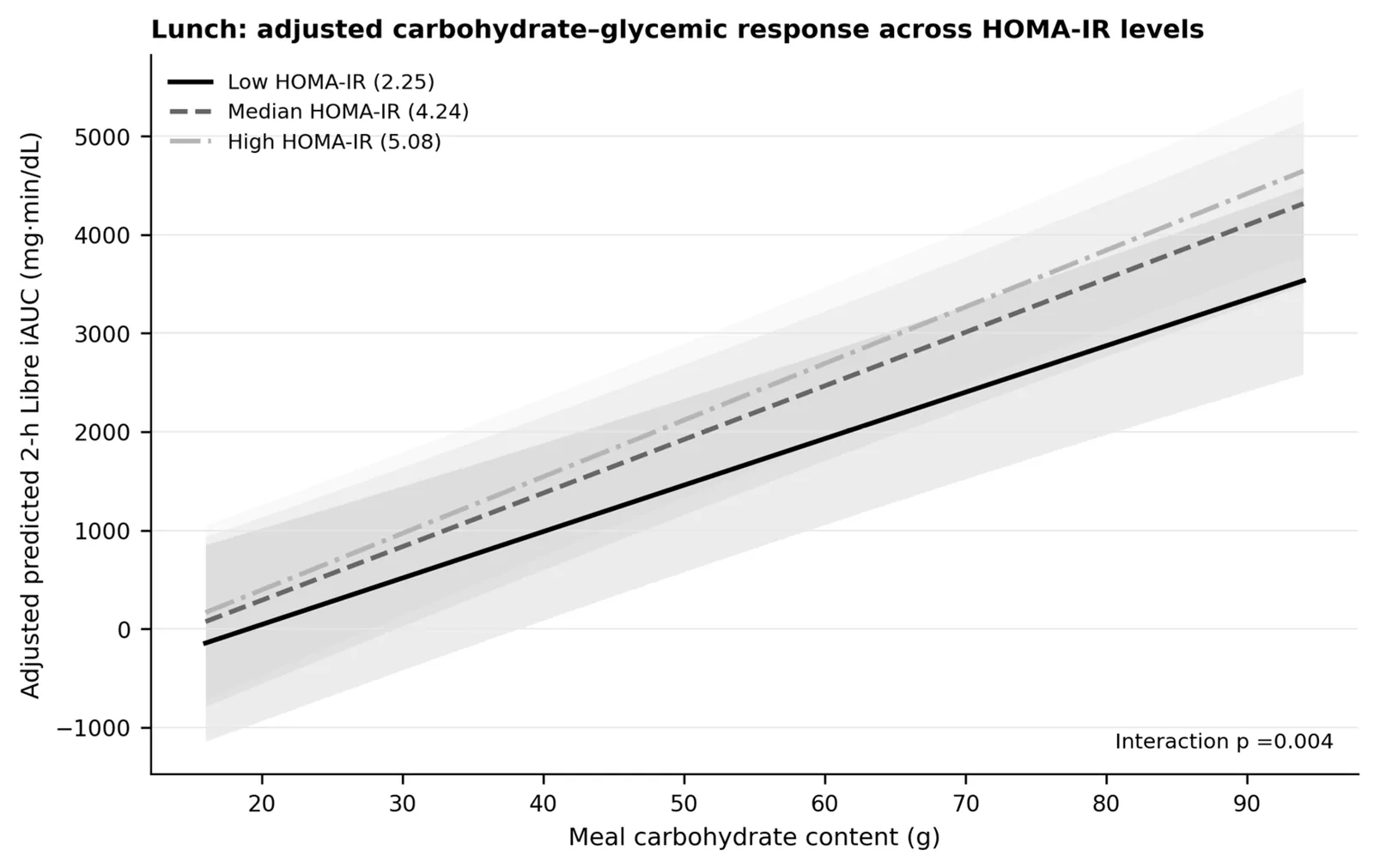
Within-cohort contextual confirmation during lunch: adjusted predicted Libre 2-h iAUC across meal carbohydrate content at low, median, and high HOMA-IR levels. Lines represent model-based estimates and shaded bands indicate 95% confidence intervals.

Adjusted prediction plots demonstrated progressively steeper carbohydrate–iAUC relationships across increasing HOMA-IR levels during both breakfast and lunch (Figure 2 and Figure 3).

### 3.3. Cross-Platform and Sensitivity Analyses

**Figure 4:**
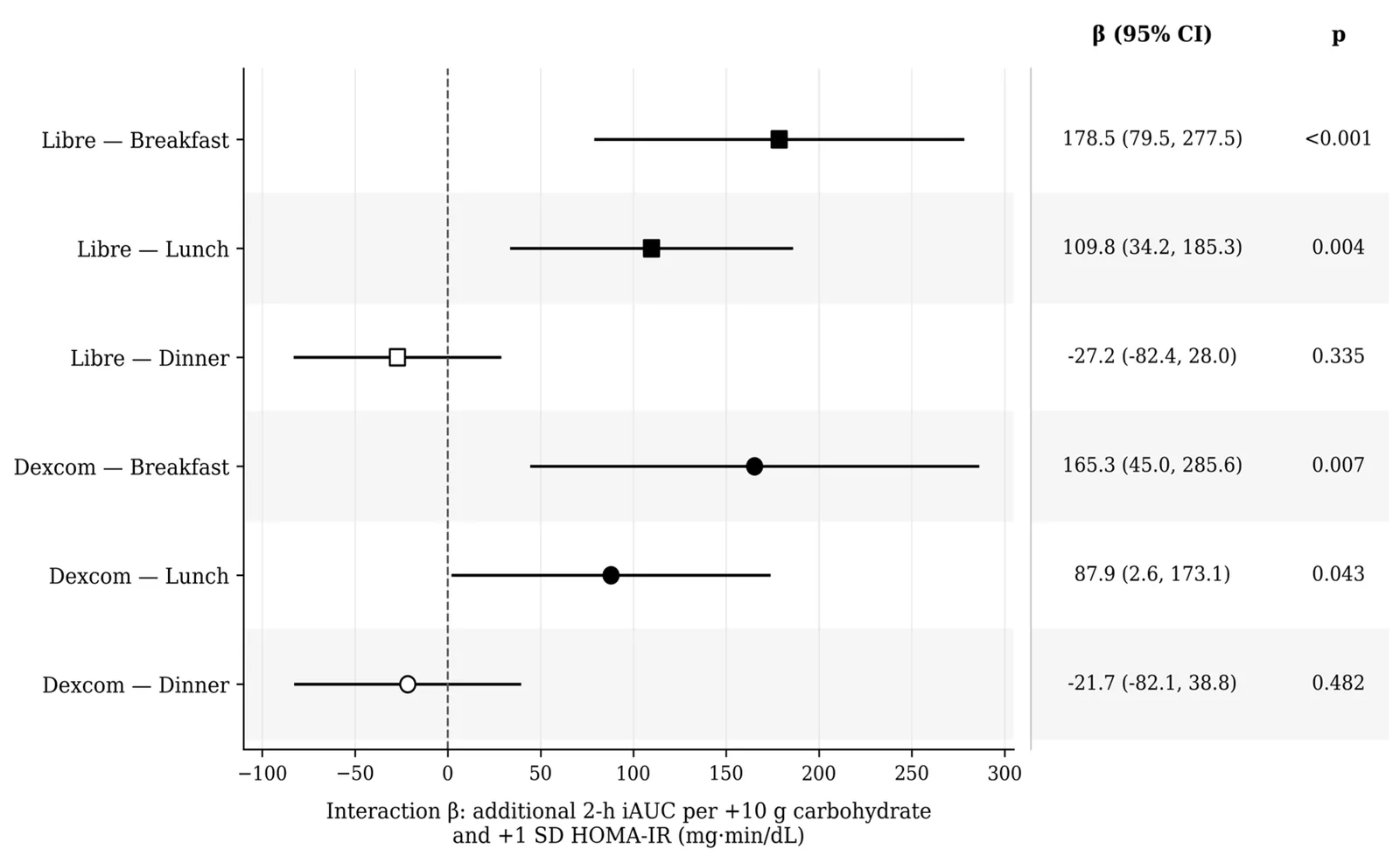
HOMA-IR × carbohydrate interaction estimates across meal contexts and CGM platforms. Squares indicate Libre and circles indicate Dexcom; filled symbols represent structured meal contexts and open symbols represent free-living dinner. Horizontal lines indicate 95% confidence intervals.

As shown in Figure 4: The direction of the HOMA-IR × carbohydrate interaction was supported using the simultaneously worn Dexcom system, with significant interactions during breakfast (β = 165.3, 95% CI 45.0–285.6; *p* = 0.007) and lunch (β = 87.9, 95% CI 2.6–173.1; *p* = 0.043). Cluster-robust GEE estimates were similarly precise in the structured contexts: Libre breakfast β = 178.0 (95% CI 92.4–263.6) and lunch β = 109.7 (52.5–166.8), and Dexcom breakfast β = 164.0 (79.7–248.2) and lunch β = 88.2 (19.1–157.2). Participant fixed-effects models also retained significance in the primary Libre analyses. When participant-specific random carbohydrate slopes were added, the Libre interaction remained evident at breakfast (β = 180.0, 95% CI 70.3–289.7; *p* = 0.001) and lunch (β = 103.0, 18.4–187.6; *p* = 0.017); Dexcom showed the same direction at breakfast (β = 166.3, 21.7–310.8; *p* = 0.024) and a less precise lunch estimate (β = 85.4, −9.8 to 180.7; *p* = 0.079). Excluding all participants with type 2 diabetes preserved the primary Libre findings at breakfast (β = 205.4, 106.5–304.2; *p* < 0.001) and lunch (β = 100.8, 17.5–184.0; *p* = 0.018). Additional adjustment for meal start time and premeal activity also preserved the Libre interactions (breakfast β = 217.3, 102.9–331.7; *p* < 0.001; lunch β = 89.3, 14.5–164.1; *p* = 0.019). Detailed sensitivity results, including less precise Dexcom secondary analyses, are provided in Table S1.

### 3.4. Comparison with Other Metabolic Effect Modifiers

HbA1c also modified the carbohydrate–glycemic response in the Libre analyses during breakfast (β = 156.2, 95% CI 55.8–256.5; *p* = 0.002; FDR-adjusted *q* = 0.014) and lunch (β = 89.3, 19.4–159.1; *p* = 0.012; *q* = 0.024). The TG/HDL-C ratio showed an interaction during breakfast (β = 129.0, 30.3–227.6; *p* = 0.010; *q* = 0.024) but not during lunch, whereas BMI showed no evidence of effect modification in either context (Table 3). In joint Libre models containing both HOMA-IR × carbohydrate and HbA1c × carbohydrate terms, the HOMA-IR interaction remained significant during breakfast (β = 138.7, 95% CI 28.1–249.3; *p* = 0.014) and lunch (β = 88.1, 2.0–174.3; *p* = 0.045), whereas HbA1c was attenuated (breakfast β = 91.9, −20.0 to 203.8; *p* = 0.107; lunch β = 49.6, −29.9 to 129.1; *p* = 0.222). In contrast, the Dexcom joint breakfast model favored HbA1c (β = 176.9, 42.3–311.6; *p* = 0.010) rather than HOMA-IR (β = 89.3, −44.0 to 222.5; *p* = 0.189), and neither marker was significant in the Dexcom lunch joint model. Thus, the marker comparison was platform-dependent and the current data do not establish universal superiority of HOMA-IR or HbA1c. Full joint-model precision estimates are provided in Table S2.

### 3.5. Free-Living Dinner Meals

In contrast to breakfast and lunch, no positive HOMA-IR-related effect modification was observed during self-selected dinner meals. The primary interaction was not significant using either Libre (β = −27.2, 95% CI −82.4 to 28.0; *p* = 0.335) or Dexcom (β = −21.7, 95% CI −82.1 to 38.8; *p* = 0.482). The corresponding 80% minimum detectable interactions were approximately 78.9 and 86.4 mg·min/dL, respectively. Thus, the dinner analyses were sufficiently precise to make a large positive interaction comparable with that observed at breakfast unlikely, although smaller effects cannot be excluded. Adjustment for pre-dinner activity, dinner start time, and the interval since the previous eating event did not materially change the null estimates. Restricting dinner carbohydrate exposure to the central common-support range across lower and higher HOMA-IR strata (0–125 g) also yielded non-positive, imprecise estimates (Libre β = −77.7, 95% CI −165.2 to 9.9; *p* = 0.082; Dexcom β = −57.6, −153.8 to 38.7; *p* = 0.241). Additional restrictions addressing extreme self-reported values and consumption completeness likewise did not reveal a reproducible positive dinner interaction (Table S4). Accordingly, the null dinner result should not be attributed to meal complexity alone; greater heterogeneity in food composition, timing and activity, differential exposure quality, and self-reporting error are competing explanations.

## 4. Discussion

In this repeated-measures analysis of CGMacros, higher insulin resistance was associated with a steeper carbohydrate–postprandial glycemia relationship in two structured meal contexts. The principal interaction was observed during breakfast and was also present during a distinct lunch context within the same cohort. The direction was supported by the second, simultaneously worn CGM device and remained evident across several alternative analytical specifications, including random-slope models and analyses excluding participants with type 2 diabetes. In contrast, a positive interaction was not evident during self-selected dinner meals. Additional dinner analyses suggested that a large positive effect comparable with the structured-meal estimates was unlikely to have been missed, while smaller effects and alternative explanations remain possible. These findings therefore support an association between host metabolic phenotype and meal carbohydrate response without establishing causality or an independent external replication.

Substantial interindividual heterogeneity in postprandial glycemic responses has been demonstrated repeatedly. Zeevi et al. showed marked variation in glycemic responses to the same foods and developed personalized prediction models integrating clinical, dietary, activity, and microbiome features [1]. The PREDICT study subsequently confirmed large interindividual variability following identical meals in more than 1000 adults and showed that both meal composition and person-specific factors contribute to postprandial glycemia [2]. The present analysis extends this literature by moving beyond prediction of response magnitude toward an interpretable physiological interaction: the glycemic effect associated with an incremental carbohydrate exposure became greater as insulin resistance increased.

This finding is particularly consistent with recent deep-phenotyping evidence. Wu et al. studied 55 participants challenged repeatedly with seven standardized carbohydrate foods and found that differential PPGR phenotypes reflected underlying metabolic physiology, including insulin resistance and beta-cell function [3]. In addition, mitigation by protein, fat, or fiber preloads was less effective in insulin-resistant participants [3]. Our findings complement those observations from a different direction. Rather than asking which standardized carbohydrate produces the highest response in a given individual, we tested whether the dose-response relationship between carbohydrate quantity and postprandial glycemia itself varies with insulin resistance. The reproducible HOMA-IR × carbohydrate interaction supports such effect modification.

The comparison with HbA1c provides additional insight but also illustrates the limits of the current sample. HbA1c modified the carbohydrate–glycemic response in both Libre breakfast and lunch analyses, consistent with the original CGMacros technical validation in which HbA1c contributed to prediction of postprandial iAUC [4]. In the joint Libre models, the HOMA-IR interaction remained statistically significant after simultaneous inclusion of the HbA1c interaction, whereas the HbA1c interaction was attenuated. However, the joint Dexcom breakfast model showed the opposite pattern, with a stronger HbA1c interaction and a non-significant HOMA-IR interaction. These platform-dependent findings should not be interpreted as evidence that either marker is universally superior. Rather, they suggest that fasting insulin-resistance phenotype and chronic average glycemia may capture overlapping but non-identical dimensions of carbohydrate susceptibility, with the relative contribution uncertain in this small cohort.

The absence of an interaction with BMI argues against a simple explanation based on adiposity alone, while TG/HDL-C showed evidence of effect modification during breakfast but not lunch. HOMA-IR showed the most consistent pattern across the primary Libre structured-meal analyses, but its physiological interpretation requires caution. HOMA-IR is calculated from fasting glucose and insulin and reflects a composite fasting state that is influenced substantially by hepatic insulin sensitivity; it is not a direct measure of postprandial skeletal-muscle glucose disposal. Clamp studies have shown moderate associations between HOMA-IR and whole-body insulin-stimulated glucose uptake while also demonstrating important contributions from basal hepatic insulin sensitivity and variation across glucose-tolerance phenotypes. Consequently, the observed HOMA-IR × carbohydrate interaction should not be interpreted as evidence that fasting or hepatic insulin resistance directly causes the postprandial response. HOMA-IR may instead index correlated physiology that includes peripheral insulin sensitivity, β-cell response, insulin secretion, and insulin clearance.

Several studies published in Metabolites also emphasize why carbohydrate grams should not be interpreted in isolation. Johansen et al. showed that a slowly digestible carbohydrate reduced postprandial glucose and insulin trajectories compared with maltodextrin across metabolically distinct populations [6]. Yong et al. demonstrated that meal timing can substantially shape the postprandial metabolome, in some analyses more strongly than glycemic index [7]. Bojarczuk et al. showed that cooking and cooling altered resistant starch and glycemic responses to chickpea pasta [8]. Together, these studies reinforce the importance of carbohydrate quality, food processing, and temporal context in determining postprandial metabolism. A recent Metabolites study also linked dietary profiles with insulin-resistance-related anthropometric and metabolic phenotypes and highlighted the need for individualized dietary approaches [9].

The dinner findings warrant a more cautious interpretation than the structured-meal results. Breakfast and lunch in CGMacros were designed to provide defined variation in macronutrient composition, whereas dinner was self-selected and recorded under free-living conditions [4]. Dinner carbohydrate exposure was not narrower overall; rather, it was substantially more heterogeneous and included extreme self-reported values. The null positive interaction persisted after adjustment for pre-dinner activity and timing and after restriction to a common central carbohydrate range, making simple lack of carbohydrate range an unlikely sole explanation. Nevertheless, food matrix, glycemic index, preparation, meal order, circadian timing, reporting error, and other unmeasured features may all alter the estimated carbohydrate slope. The dinner result should therefore be viewed as a context-specific null finding with multiple plausible explanations, not as proof that meal complexity itself abolishes insulin-resistance-related effect modification.

The cross-device results also deserve consideration. Simultaneous CGM devices can yield materially different postprandial estimates; Howard et al. demonstrated discordant meal rankings between two concurrently worn CGM systems in adults without diabetes [10], and CGMacros reported systematic differences in absolute glucose measurements between Libre and Dexcom [4]. Preservation of the direction and approximate magnitude of the primary interaction across devices therefore provides useful cross-device robustness. However, Libre and Dexcom were worn simultaneously during the same meals and do not constitute independent replication cohorts. Several Dexcom secondary analyses were also less precise, including the random-slope lunch model. Libre should therefore remain the primary platform, with Dexcom interpreted as supportive device-level robustness rather than independent validation.

The findings may have implications for precision nutrition, but their current role is hypothesis-generating. Personalized postprandial glucose response-targeting diets have improved glycemic control in prediabetes in randomized studies [11], while personalized dietary approaches have also been evaluated in adults with abnormal glucose metabolism and obesity [12,13]. Our results suggest one potentially interpretable component of such personalization: the glycemic consequence associated with an additional amount of carbohydrate may differ according to the individual’s metabolic phenotype. However, this observational secondary analysis [14] cannot establish that carbohydrate prescriptions or thresholds should be based on HOMA-IR, and prospective intervention studies with external validation and direct measures of insulin sensitivity are required before clinical translation.

This study has several strengths. The repeated-measures structure provided multiple meal challenges within each participant and enabled participant-level modeling rather than treating meals as independent observations. The principal interaction was observed during breakfast and was also evident in a distinct structured lunch context within the same cohort. Two simultaneously worn CGM devices allowed assessment of cross-device robustness. The primary Libre finding remained evident after adjustment for postprandial and premeal activity, meal timing, restriction to fully consumed meals, participant fixed-effects modeling, GEE analysis, a participant-specific random carbohydrate slope, and exclusion of participants with type 2 diabetes. Detailed exposure-overlap analyses further showed substantial within-person carbohydrate variation and comparable structured-meal carbohydrate ranges across lower and higher HOMA-IR strata.

Several limitations should be acknowledged. First, the cohort contained only 45 participants, with 44 contributing to the iAUC analytic sets. Repeated meals improve estimation of within-participant meal-response relationships but do not replace a larger number of independent individuals, particularly for an interaction involving the between-participant HOMA-IR phenotype; confidence intervals and the secondary-device analyses therefore remain important indicators of uncertainty. Second, HOMA-IR is a fasting surrogate rather than a clamp-derived measure of peripheral insulin sensitivity, and its interpretation may vary with fasting glycemia and β-cell function [15,16]. Third, the original protocol excluded insulin, injectable GLP-1 receptor agonists, and oral agents other than metformin in participants with type 2 diabetes [4], but participant-level metformin use was unavailable in the public data. Fourth, caffeine intake, intra-meal food sequence, sleep, and detailed circadian measures were not available for adjustment. Meal start time and premeal activity analyses partly address temporal and activity-related confounding but cannot eliminate these residual factors. Fifth, dinner intake was self-reported and contained substantially greater exposure heterogeneity than the structured meals, increasing susceptibility to reporting and food-composition error. Finally, CGM systems measure interstitial rather than plasma glucose and can differ systematically in absolute values and meal ranking [10].

## 5. Conclusions

In this observational repeated-measures analysis, insulin resistance modified the glycemic impact of carbohydrate across two structured meal contexts, with supportive evidence from a second simultaneously worn CGM device and multiple robustness analyses. The findings identify an interpretable association between host metabolic phenotype and meal carbohydrate exposure but should be considered hypothesis-generating rather than prescriptive. The absence of a reproducible positive interaction during heterogeneous self-selected dinners indicates that the structured-meal result cannot be assumed to generalize unchanged to free-living meals. Larger prospective studies using direct measures of insulin sensitivity, independent cohorts, and more complete characterization of meal timing and composition are required before insulin-resistance phenotype can be used to guide individualized carbohydrate recommendations.

## Figures and Tables

**Table 1:** Baseline characteristics of the study population according to glycemic status.

Variable	Overall	Normoglycemia	Prediabetes	Diabetes	*p*-Value
Age, years	51.00 [40.00–58.00]	40.00 [25.50–48.00]	54.00 [45.75–58.25]	52.50 [51.00–59.00]	0.006
Female sex, n (%)	29 (64.4%)	10 (66.7%)	11 (68.8%)	8 (57.1%)	0.784
BMI, kg/m^2^	30.04 [26.92–35.92]	25.80 [23.02–30.49]	32.76 [28.83–36.22]	30.62 [28.24–34.35]	0.069
HbA1c, %	5.90 [5.50–6.90]	5.30 [5.05–5.45]	5.95 [5.80–6.12]	7.10 [6.93–7.35]	<0.001
Fasting glucose, mg/dL	109.00 [100.00–142.00]	96.00 [90.50–101.00]	110.50 [104.00–125.25]	149.00 [142.50–157.00]	<0.001
Fasting insulin, µU/mL	13.50 [9.30–17.80]	9.30 [5.25–13.20]	17.50 [13.03–20.27]	13.20 [10.55–17.48]	0.014
HOMA-IR	4.24 [2.25–5.08]	1.99 [1.11–3.36]	4.61 [3.74–5.52]	5.06 [4.03–6.67]	<0.001
Triglycerides, mg/dL	121.00 [83.00–154.00]	83.00 [64.00–122.00]	120.00 [98.00–148.50]	160.50 [124.50–248.00]	0.008
HDL-C, mg/dL	51.00 [42.00–60.00]	57.00 [51.50–65.50]	46.50 [39.75–52.75]	43.50 [38.00–57.50]	0.018
TG/HDL ratio	2.14 [1.64–4.64]	1.57 [1.03–2.15]	2.45 [1.90–3.62]	3.76 [2.04–6.08]	0.005

Values are median [IQR] unless otherwise stated. Continuous variables were compared using the Kruskal–Wallis test and sex using the Pearson chi-square test. BMI, body mass index; HDL-C, high-density lipoprotein cholesterol; HOMA-IR, homeostasis model assessment of insulin resistance; TG, triglycerides.

**Table 2:** HOMA-IR × carbohydrate interaction across meal contexts and CGM platforms.

CGM	Meal	Sample	Interaction β	95% CI	Mixed *p*	GEE *p*
Libre	Breakfast	423 meals/44 participants	178.5	79.5 to 277.5	<0.001	<0.001
Libre	Lunch	414 meals/44 participants	109.8	34.2 to 185.3	0.004	<0.001
Libre	Dinner	372 meals/44 participants	−27.2	−82.4 to 28.0	0.335	0.378
Dexcom	Breakfast	394 meals/44 participants	165.3	45.0 to 285.6	0.007	<0.001
Dexcom	Lunch	410 meals/44 participants	87.9	2.6 to 173.1	0.043	0.012
Dexcom	Dinner	370 meals/44 participants	−21.7	−82.1 to 38.8	0.482	0.464

Interaction β represents the additional change in 2-h iAUC (mg·min/dL) per +10 g carbohydrate for each +1 SD HOMA-IR. Mixed-effects models included a participant random intercept and were adjusted for protein, fat, fiber, premeal glucose, age, sex, and BMI. GEE models used an exchangeable working correlation clustered by participant.

**Table 3:** Comparison of metabolic modifiers of the carbohydrate–glycemic response in the primary Libre analyses.

Modifier	Meal	Interaction β	95% CI	*p*	BH-FDR *q*
HOMA-IR	Breakfast	178.5	79.5 to 277.5	<0.001	Primary hypothesis
HOMA-IR	Lunch	109.8	34.2 to 185.3	0.004	Primary hypothesis
HbA1c	Breakfast	156.2	55.8 to 256.5	0.002	0.014
HbA1c	Lunch	89.3	19.4 to 159.1	0.012	0.024
TG/HDL	Breakfast	129.0	30.3 to 227.6	0.010	0.024
TG/HDL	Lunch	40.9	−28.5 to 110.3	0.248	0.372
BMI	Breakfast	−2.1	−103.5 to 99.3	0.968	0.968
BMI	Lunch	−24.1	−98.4 to 50.3	0.526	0.631

Interaction β is expressed as the additional change in 2-h iAUC (mg·min/dL) per +10 g carbohydrate for each +1 SD higher metabolic marker. HOMA-IR was the prespecified primary exposure and was not included in the false-discovery-rate correction. Benjamini–Hochberg correction was applied to the six secondary tests for HbA1c, TG/HDL-C, and BMI across breakfast and lunch.

## Data Availability

The data analyzed in this study are publicly available in PhysioNet as the CGMacros dataset (version 1.0.0; DOI: 10.13026/3z8q-x658) [14]. The derived analysis-ready datasets and statistical code can be made available by the corresponding author upon reasonable request.

## Supplementary Materials

The following supporting information can be downloaded at: https://www.mdpi.com/article/10.3390/metabo16090671/s1, Table S1: Sensitivity and secondary analyses of the HOMA-IR × carbohydrate interaction; Table S2: Joint HOMA-IR and HbA1c interaction models; Table S3: Meal composition, carbohydrate exposure, and overlap across HOMA-IR strata; Table S4: Dinner-specific sensitivity and precision analyses.

## Abbreviations

The following abbreviations are used in this manuscript:

BMI	body mass index
CGM	continuous glucose monitoring
GEE	generalized estimating equations
HbA1c	glycated hemoglobin
HDL-C	high-density lipoprotein cholesterol
HOMA-IR	homeostasis model assessment of insulin resistance
iAUC	incremental area under the curve
IQR	interquartile range
MET	metabolic equivalent of task
PPGR	postprandial glycemic response
T2D	type 2 diabetes
TG	triglycerides

## References

[1] Zeevi D., Korem T., Zmora N., Israeli D., Rothschild D., Weinberger A., Ben-Yacov O., Lador D., Avnit-Sagi T., Lotan-Pompan M. (2015). Personalized Nutrition by Prediction of Glycemic Responses. Cell.

[2] Berry S.E., Valdes A.M., Drew D.A., Asnicar F., Mazidi M., Wolf J., Capdevila J., Hadjigeorgiou G., Davies R., Al Khatib H. (2020). Human Postprandial Responses to Food and Potential for Precision Nutrition. Nat. Med..

[3] Wu Y., Ehlert B., Metwally A.A., Perelman D., Park H., Brooks A.W., Abbasi F., Michael B., Celli A., Bejikian C. (2025). Individual Variations in Glycemic Responses to Carbohydrates and Underlying Metabolic Physiology. Nat. Med..

[4] Das A., Kerr D., Glantz N., Bevier W., Santiago R., Gutierrez-Osuna R., Mortazavi B.J. (2025). CGMacros: A Pilot Scientific Dataset for Personalized Nutrition and Diet Monitoring. Sci. Data.

[5] Matthews D.R., Hosker J.P., Rudenski A.S., Naylor B.A., Treacher D.F., Turner R.C. (1985). Homeostasis Model Assessment: Insulin Resistance and Beta-Cell Function from Fasting Plasma Glucose and Insulin Concentrations in Man. Diabetologia.

[6] Johansen O.E., Neutel J., Gupta S., Mariani B., Ufheil G., Perrin E., Rytz A., Lahiry A., Delodder F., Lerea-Antes J. (2024). Oligomalt, a New Slowly Digestible Carbohydrate, Reduces Post-Prandial Glucose and Insulin Trajectories Compared to Maltodextrin across Different Population Characteristics: Double-Blind Randomized Controlled Trials in Healthy Individuals, People with Obesity, and People with Type 2 Diabetes. Metabolites.

[7] Yong Y.N., Dong J., Pakkiri L.S., Henry C.J., Haldar S., Drum C.L. (2023). Chronometabolism: The Timing of the Consumption of Meals Has a Greater Influence Than Glycemic Index (GI) on the Postprandial Metabolome. Metabolites.

[8] Bojarczuk A., Kęszycka P., Marszałek K., Gajewska D. (2024). The Effect of Cooking and Cooling Chickpea Pasta on Resistant Starch Content, Glycemic Response, and Glycemic Index in Healthy Adults. Metabolites.

[9] Wiśniewska-Ślepaczuk K., Żak-Kowalska K., Moskal A., Kowalski S., Al-Wathinani A.M., Alhajlah M., Goniewicz K., Goniewicz M. (2024). Nutritional Profiles and Their Links to Insulin Resistance and Anthropometric Variables in a Female Cohort. Metabolites.

[10] Howard R., Guo J., Hall K.D. (2020). Imprecision Nutrition? Different Simultaneous Continuous Glucose Monitors Provide Discordant Meal Rankings for Incremental Postprandial Glucose in Subjects without Diabetes. Am. J. Clin. Nutr..

[11] Ben-Yacov O., Godneva A., Rein M., Shilo S., Kolobkov D., Koren N., Dolev N.C., Shmul T.T., Wolf B.C., Kosower N. (2021). Personalized Postprandial Glucose Response-Targeting Diet Versus Mediterranean Diet for Glycemic Control in Prediabetes. Diabetes Care.

[12] Popp C.J., Hu L., Kharmats A.Y., Curran M., Berube L.T., Wang C., Pompeii M.L., Illiano P., St-Jules D.E., Mottern M. (2022). Effect of a Personalized Diet to Reduce Postprandial Glycemic Response vs a Low-Fat Diet on Weight Loss in Adults with Abnormal Glucose Metabolism and Obesity: A Randomized Clinical Trial. JAMA Netw. Open.

[13] Bermingham K.M., Linenberg I., Polidori L., Asnicar F., Arrè A., Wolf J., Badri F., Bernard H., Capdevila J., Bulsiewicz W.J. (2024). Effects of a Personalized Nutrition Program on Cardiometabolic Health: A Randomized Controlled Trial. Nat. Med..

[14] Gutierrez-Osuna R., Kerr D., Mortazavi B., Das A. (2025). CGMacros: A Scientific Dataset for Personalized Nutrition and Diet Monitoring, version 1.0.0.

[15] Tripathy D., Almgren P., Tuomi T., Groop L. (2004). Contribution of Insulin-Stimulated Glucose Uptake and Basal Hepatic Insulin Sensitivity to Surrogate Measures of Insulin Sensitivity. Diabetes Care.

[16] Meyer C., Pimenta W., Woerle H.J., Van Haeften T., Szoke E., Mitrakou A., Gerich J. (2006). Different Mechanisms for Impaired Fasting Glucose and Impaired Postprandial Glucose Tolerance in Humans. Diabetes Care.

