# Supplementary material for: Insulin Resistance Modifies the Glycemic Impact of Carbohydrate Across Distinct Meal Contexts: A Repeated-Measures Dual-CGM Study
Source: Metabolites. 2026 Sep 11;16(9):671. doi: 10.3390/metabo16090671 (PMC13608909; doi:10.3390/metabo16090671)
Supplement: Supplementary file 1 [file metabolites-16-00671-s001.zip › metabolites-4544322-Supplementary.pdf]

Supplementary Materials

Insulin Resistance Modifies the Glycemic Impact of Carbohydrate Across Distinct Meal Contexts: A Repeated-Measures Dual-CGM Study

Buket Yilmaz Bulbul, Burak Andac, and Mehmet Celik

Table S1. Sensitivity and secondary analyses of the HOMA-IR × carbohydrate interaction.

CGM	Meal	Analysis	Sample	Interaction effect (95% CI)	p
Libre	Breakfast	Primary mixed model	423 meals / 44 participants	178.5 (79.5 to 277.5)	<0.001
Libre	Breakfast	+ postmeal activity	318 meals / 33 participants	218.7 (104.6 to 332.7)	<0.001
Libre	Breakfast	100% consumed meals	403 meals / 42 participants	189.2 (87.9 to 290.4)	<0.001
Libre	Breakfast	Subject fixed effects	423 meals / 44 participants	182.6 (91.7 to 273.5)	<0.001
Libre	Breakfast	Peak glucose outcome	423 meals / 44 participants	2.0 (0.9 to 3.1)	<0.001
Libre	Lunch	Primary mixed model	414 meals / 44 participants	109.8 (34.2 to 185.3)	0.004
Libre	Lunch	+ postmeal activity	310 meals / 33 participants	90.6 (15.9 to 165.4)	0.017
Libre	Lunch	100% consumed meals	355 meals / 42 participants	88.5 (8.0 to 169.0)	0.031
Libre	Lunch	Subject fixed effects	414 meals / 44 participants	116.8 (52.9 to 180.7)	<0.001
Libre	Lunch	Peak glucose outcome	414 meals / 44 participants	0.8 (-0.1 to 1.7)	0.065
Dexcom	Breakfast	Primary mixed model	394 meals / 44 participants	165.3 (45.0 to 285.6)	0.007
Dexcom	Breakfast	+ postmeal activity	296 meals / 33 participants	146.6 (13.8 to 279.5)	0.031
Dexcom	Breakfast	100% consumed meals	377 meals / 42 participants	174.6 (53.4 to 295.7)	0.005
Dexcom	Breakfast	Subject fixed effects	394 meals / 44 participants	174.2 (77.2 to 271.3)	<0.001
Dexcom	Breakfast	Peak glucose outcome	394 meals / 44 participants	1.9 (0.4 to 3.3)	0.011

Table S1. (continued)

CGM	Meal	Analysis	Sample	Interaction effect (95% CI)	p
Dexcom	Lunch	Primary mixed model	410 meals / 44 participants	87.9 (2.6 to 173.1)	0.043
Dexcom	Lunch	+ postmeal activity	308 meals / 33 participants	60.1 (-24.3 to 144.4)	0.163
Dexcom	Lunch	100% consumed meals	351 meals / 42 participants	66.2 (-21.8 to 154.2)	0.140
Dexcom	Lunch	Subject fixed effects	410 meals / 44 participants	97.3 (18.4 to 176.1)	0.016
Dexcom	Lunch	Peak glucose outcome	410 meals / 44 participants	1.1 (0.0 to 2.1)	0.042

Notes: For iAUC analyses, the interaction effect is the additional change in 2-h iAUC (mg·min/dL) per +10 g meal carbohydrate for each +1 SD higher HOMA-IR. For peak glucose analyses, the interaction effect is expressed in mg/dL. Primary mixed-effects models included a participant-specific random intercept and were adjusted for protein, fat, fiber, premeal glucose, age, sex, and BMI. Activity-adjusted models additionally included mean postprandial METs. "100% consumed meals" analyses were restricted to fully consumed meals. Participant fixed-effects models control for all participant characteristics that are constant across meals.

Table S1. (continued)

CGM	Meal	Analysis	Sample	Interaction effect (95% CI)	p
Libre	Breakfast	GEE with cluster-robust SE	423 meals / 44 participants	178.0 (92.4 to 263.6)	<0.001
Libre	Lunch	GEE with cluster-robust SE	414 meals / 44 participants	109.7 (52.5 to 166.8)	<0.001
Dexcom	Breakfast	GEE with cluster-robust SE	394 meals / 44 participants	164.0 (79.7 to 248.2)	<0.001
Dexcom	Lunch	GEE with cluster-robust SE	410 meals / 44 participants	88.2 (19.1 to 157.2)	0.012
Libre	Breakfast	Random intercept + carbohydrate random slope	423 meals / 44 participants	180.0 (70.3 to 289.7)	0.001
Libre	Lunch	Random intercept + carbohydrate random slope	414 meals / 44 participants	103.0 (18.4 to 187.6)	0.017
Dexcom	Breakfast	Random intercept + carbohydrate random slope	394 meals / 44 participants	166.3 (21.7 to 310.8)	0.024
Dexcom	Lunch	Random intercept + carbohydrate random slope	410 meals / 44 participants	85.4 (-9.8 to 180.7)	0.079
Libre	Breakfast	Excluding participants with type 2 diabetes	290 meals / 30 participants	205.4 (106.5 to 304.2)	<0.001
Libre	Lunch	Excluding participants with type 2 diabetes	276 meals / 30 participants	100.8 (17.5 to 184.0)	0.018
Dexcom	Breakfast	Excluding participants with type 2 diabetes	271 meals / 30 participants	112.4 (-11.1 to 235.8)	0.074
Dexcom	Lunch	Excluding participants with type 2 diabetes	272 meals / 30 participants	77.3 (-18.4 to 173.0)	0.113

CGM	Meal	Analysis	Sample	Interaction effect (95% CI)	p
Libre	Breakfast	+ meal start time and premeal activity	318 meals / 33 participants	217.3 (102.9 to 331.7)	<0.001
Libre	Lunch	+ meal start time and premeal activity	310 meals / 33 participants	89.3 (14.5 to 164.1)	0.019

Notes: GEE models used an exchangeable working correlation clustered by participant. Random-slope models included a participant-specific random intercept and a random slope for mean-centered carbohydrate. The type 2 diabetes exclusion analysis re-standardized HOMA-IR within the restricted participant sample. Meal start-time analyses additionally adjusted for mean activity during the 60 min preceding the meal. Interaction effects are expressed as additional 2-h iAUC (mg·min/dL) per +10 g carbohydrate and +1 SD higher HOMA-IR.

Table S2. Joint HOMA-IR and HbA1c interaction models.

CGM	Meal	HOMA-IR interaction β (95% CI; p)	HbA1c interaction β (95% CI; p)
Libre	Breakfast	138.7 (28.1 to 249.3; p=0.014)	91.9 (−20.0 to 203.8; p=0.107)
Libre	Lunch	88.1 (2.0 to 174.3; p=0.045)	49.6 (−29.9 to 129.1; p=0.222)
Dexcom	Breakfast	89.3 (−44.0 to 222.5; p=0.189)	176.9 (42.3 to 311.6; p=0.010)
Dexcom	Lunch	76.6 (−20.3 to 173.4; p=0.121)	31.7 (−57.7 to 121.0; p=0.487)

Notes: HOMA-IR and HbA1c were standardized before modeling. Both carbohydrate × HOMA-IR and carbohydrate × HbA1c interaction terms were entered simultaneously. Interaction β values represent the additional 2-h iAUC (mg·min/dL) per +10 g carbohydrate and +1 SD higher metabolic marker; p values are shown in parentheses. Models otherwise used the same covariate structure as the primary analysis.

Table S3. Meal composition, carbohydrate exposure, and overlap across HOMA-IR strata.

Panel A. Meal composition in the primary Libre analytic sets.

Meal	Sample	Carbohydrate, g	Protein, g	Fat, g	Fiber, g	Energy, kcal	Carbohydrate 5th–95th percentile, g
Breakfast	423 meals / 44 participants	66 [66–66] (24–73)	22 [22–66]	10.5 [10.5–42.0]	0 [0–0]	608 [448–712]	24.0–73.0
Lunch	414 meals / 44 participants	76 [40–93] (16–94)	38 [20–76]	20.0 [15.0–42.0]	10 [4–13]	585 [445–830]	16.0–94.0
Dinner	372 meals / 44 participants	44 [22–70] (0–761)	20 [6–38]	13.0 [5.8–28.2]	4 [1–8]	420 [220–681]	0.0–131.9

Panel B. Carbohydrate exposure by HOMA-IR stratum.

Meal	HOMA-IR stratum	Meals	Participants	Carbohydrate, g	5th–95th percentile, g
Breakfast	Low (≤Q1)	116	12	66.0 [66.0–66.0] (24–73)	24.0–73.0
Breakfast	Middle	189	20	66.0 [66.0–66.0] (24–73)	24.0–73.0
Breakfast	High (≥Q3)	118	12	66.0 [66.0–66.0] (24–73)	24.0–73.0

Meal	HOMA-IR stratum	Meals	Participants	Carbohydrate, g	5th–95th percentile, g
Lunch	Low (≤Q1)	112	12	78.5 [40.0–93.0] (16–94)	16.0–94.0
Lunch	Middle	190	20	78.5 [40.0–93.0] (16–94)	16.0–94.0
Lunch	High (≥Q3)	112	12	76.0 [40.0–93.0] (16–94)	16.0–94.0
Dinner	Low (≤Q1)	100	12	48.0 [24.8–81.2] (0–761)	0.0–131.1
Dinner	Middle	173	20	47.0 [24.0–66.0] (0–204)	0.0–127.6
Dinner	High (≥Q3)	99	12	38.0 [16.0–63.0] (0–190)	0.0–124.9

Notes: Values are median [IQR] (range) unless otherwise stated. Lower HOMA-IR was defined as ≤ the cohort 25th percentile (2.25) and higher HOMA-IR as ≥ the 75th percentile (5.08). The structured breakfast and lunch contexts showed nearly identical carbohydrate ranges across lower and higher HOMA-IR strata. Median within-participant carbohydrate ranges were 49 g at breakfast, 78 g at lunch, and 82.5 g at dinner.

Table S4. Dinner-specific sensitivity and precision analyses.

Device	Analysis	Meals	Participants	Effect (95% CI)	p
Libre	Primary model	372	44	-27.2 (-82.4 to 28.0)	0.335
Libre	Carbohydrate ≤ context 95th percentile	353	44	-74.7 (-161.5 to 12.1)	0.092
Libre	Known fully consumed meals (amount consumed = 1 or 100)	196	35	-42.4 (-184.5 to 99.6)	0.558
Libre	+ mean activity during 120 min before dinner	289	33	-32.3 (-90.4 to 25.9)	0.277
Libre	+ dinner start time and interval since previous eating event	372	44	-31.2 (-85.6 to 23.1)	0.26
Libre	+ pre-dinner activity, start time, and previous-event interval	289	33	-33.9 (-91.3 to 23.4)	0.246
Dexcom	Primary model	370	44	-21.7 (-82.1 to 38.8)	0.482
Dexcom	Carbohydrate ≤ context 95th percentile	351	44	-55.1 (-150.5 to 40.3)	0.257
Dexcom	Known fully consumed meals (amount consumed = 1 or 100)	194	35	-79.7 (-232.5 to 73.2)	0.307

Device	Analysis	Meals	Participants	Effect (95% CI)	p
Dexcom	+ mean activity during 120 min before dinner	288	33	-33.3 (-97.2 to 30.5)	0.306
Dexcom	+ dinner start time and interval since previous eating event	370	44	-24.7 (-83.8 to 34.4)	0.413
Dexcom	+ pre-dinner activity, start time, and previous-event interval	288	33	-31.8 (-94.3 to 30.8)	0.32
Libre	Central common carbohydrate support (0–125 g)	352	44	-77.7 (-165.2 to 9.9)	0.082
Libre	Stricter data-dictionary plausibility bounds	282	43	-48.8 (-153.7 to 56.1)	0.362
Dexcom	Central common carbohydrate support (0–125 g)	350	44	-57.6 (-153.8 to 38.7)	0.241
Dexcom	Stricter data-dictionary plausibility bounds	280	43	-21.3 (-138.4 to 95.8)	0.721

Notes: The primary dinner interaction was −27.2 (95% CI −82.4 to 28.0) with Libre and −21.7 (−82.1 to 38.8) with Dexcom. The corresponding two-sided α=0.05 80% minimum detectable interaction magnitudes were approximately 78.9 and 86.4 mg·min/dL. The central common-support restriction (0–125 g carbohydrate) was based on the overlapping 5th–95th percentile range across lower and higher HOMA-IR strata. The stricter plausibility analysis restricted macronutrient and amount-consumed values to the ranges described in the accompanying PhysioNet data dictionary. None of the dinner sensitivity analyses demonstrated a reproducible positive HOMA-IR × carbohydrate interaction across both devices.
